# Antibacterial Activity of Biocellulose with Oregano Essential Oil against *Cronobacter* Strains

**DOI:** 10.3390/polym12081647

**Published:** 2020-07-24

**Authors:** Gulden Nagmetova, Anna Berthold-Pluta, Monika Garbowska, Askar Kurmanbayev, Lidia Stasiak-Różańska

**Affiliations:** 1 National Center for Biotechnology, Laboratory of Ecological Biotechnology, 13/5, Kurgalzhynskoye Road, Nur-Sultan 010000, Kazakhstan; gulden30-04@mail.ru (G.N.); kurmanbayev@biocenter.kz (A.K.); 2 Department of General Biology and Genomics, L.N. Gumilyov Eurasian National University, Kazhymukan 13 St., Nur-Sultan 010000, Kazakhstan; 3 Division of Milk Technology, Department of Food Technology and Assessment, Institute of Food Sciences, Warsaw University of Life Sciences—SGGW, Nowoursynowska 159C St., 02-787 Warsaw, Poland; anna_berthold_pluta@sggw.edu.pl (A.B.-P.); monika_garbowska@sggw.edu.pl (M.G.)

**Keywords:** biocellulose, *Cronobacter*, antibacterial activity, oregano essential oil, food packaging

## Abstract

Biocellulose, named “the biomaterial of the future”, is a natural and ecologically friendly polymer, produced by selected acetic acid bacteria strains. Biocellulose impregnated with antimicrobial agents can be used as a novel, safe, and biodegradable food packaging material, helping extend the shelf life of some products and may also have the chance to replace typical plastic packaging, which is a big environmental problem these days. This study aimed to evaluate if cellulose impregned with natural oregano essential oil could show antibacterial activity against *Cronobacter* strains, which can occur in food, causing diseases and food poisoning. Bacterial cellulose was obtained from two acetic bacteria strains, *Gluconacetobacter hansenii* ATCC 23769 and *Komagataeibacter* sp. GH1. Antibacterial activity was studied by the disc-diffusion method against chosen *Cronobacter* strains, isolated from the plant matrix. Oregano essential oil has been shown to penetrate into the structure of bacterial cellulose, and after applying cellulose to the solid medium, it showed the ability to migrate. Biopolymer from the strain *K*. sp. GH1 was able to better absorb and retain essential oregano oil (OEO) compared to bacterial cellulose (BC) produced by the *G. hansenii* ATCC 23769. Bacterial cellulose with oregano essential oil from strain *Komagataeibacter* GH1 showed generally greater inhibitory properties for the growth of tested strains than its equivalent obtained from *G. hansenii*. This was probably due to the arrangement of the polymer fibers and its final thickness. The largest zone of inhibition of strain growth was observed in relation to *C. condimenti* s37 (32.75 mm ± 2.8). At the same time, the control sample using filter paper showed an inhibition zone of 36.0 mm ± 0.7. A similar inhibition zone (28.33 mm ± 2.6) was observed for the *C. malonaticus* lv31 strain, while the zone in the control sample was 27.1 mm ± 0.7. Based on this study, it was concluded that bacterial cellulose impregnated with oregano essential oil has strong and moderate antimicrobial activity against all presented strains of the genus *Cronobacter* isolated from plant matrix. Obtained results give a strong impulse to use this biopolymer as ecological food packaging in the near future.

## 1. Introduction

Biocellulose, also known as bacterial cellulose (BC), is one of the natural and environmentally friendly biopolymer produced by selected species of acetic acid bacteria and is a promising nano-biomaterial with unique properties [1,2].

It was shown that BC can be synthesized by various microorganisms as a porous material with high permeability to liquids and gases as well as high ability to absorb water (water content > 90%) [3,4]. The unique physical and mechanical properties of BC as well as chemical purity [5] served as the basis for its application in various fields which can be in the creation of high-quality audio membranes, electronic paper [6], or the production of dietary fiber and gelling substance in the food industry [7].

Bacterial cellulose is a non-toxic material, which is biocompatible with human and animal skin [8,9]. Fibrils of bacterial cellulose are 100 times thinner than in plant cellulose, creating a highly porous material that allows for the transfer of antibiotics or other medicines into the wound, while remaining an effective physical barrier against any external infections. BC is non-allergenic material, which can be easily sterilized without changing properties. It can be used as a substitute for skin in the treatment of extensive burns and non-woven coatings for chronic wounds. For this reason, cellulose is widely used to treat wounds [10].

Many researchers have studied the use of BC as a biological non-woven material with special emphasis on the use of packaging made from natural and biodegradable materials to reduce the use of synthetic materials that contribute to environmental pollution [11,12] and to extend the shelf life of food products [13].

Active packaging including antimicrobial ones is a type of packaging that has attracted the attention of both researchers and industry, and today has numerous commercial applications. Antimicrobial agents can be released by evaporation or enter food through diffusion and distribution, directly when applied to food surfaces, or indirectly when incorporated into carrier materials such as coatings or food wraps [14].

Natural or chemical antimicrobial compounds can be used to create active packaging based on antimicrobial properties. Due to the fact that using chemicals in food raises consumer concerns, extensive efforts are being made to exclude their use and replace them by natural ones, which show antimicrobial activity and can extend the shelf life of some products [15].

Essential oils are complex mixtures of strongly active compounds that are very volatile and sensitive to light, oxygen, moisture, and temperature. Loading inside nanocarriers can be a strategy to increase their stability and successfully use them in therapies and in industry [16]. There is significant research showing that essential oils extracted from plants have antibacterial effects. It was shown that wormwood essential oil has antibacterial activity against, among others, *Candida parapsilosis*, *Enterococcus faecium*, *Escherichia coli*, *Pseudomonas aeruginosa*, or *Staphylococcus aureus* [17]. Additionally, antimicrobial assays demonstrated that Pompia (citrus species belonging to Sardinian endemic biodiversity) and lemon juices have inhibitory and antibiofilm effects against chosen pathogenic bacteria such as *P. aeruginosa*, *S. aureus*, and *Enterococcus faecalis* [18].

One of the substance exhibiting antibacterial activity is oregano essential oil (OEO) [19]. Oregano oil is extracted from the oregano plant (*Origanum vulgare*), a perennial herb from the flowering plant family *Lamiaceae*. Oregano is an aromatic plant with decorative, culinary, and phytotherapeutic applications worldwide. In Europe, it is used traditionally in southern countries, especially, in the Mediterranean region. The antimicrobial activity of essential oils obtained from these plants has attracted the attention of scientists, as they can be used as an alternative to the growing resistance of traditional antibiotics to diseases caused by pathogens [20]. Oregano oil, used as a food flavoring, has a wide spectrum of antimicrobial activity due to its high content of phenolic derivatives such as carvacrol and thymol [21]. There are many scientific reports showing the chemical composition and antimicrobial properties of essential oils of various types of origanum and their use in various commercial preparations as antimicrobial and antioxidant agents [22].

Bacteria genus *Cronobacter* spp. have been isolated from food and environmental, human, and clinical sources. *Cronobacter* can occur in a wide variety of foods including plant materials, infant formula, cheese, dried foods and meats as well as water. [23]. The genus of *Cronobacter* has been associated with rare but life-threatening diseases mainly in newborns and infants including meningitis, septicemia, and necrotizing enterocolitis [24,25], but also in the elderly and immunocompromised adults including pneumonia, septicemia, osteomyelitis, splenic abscesses, and wound infections [26,27]. According to the molecular identification, the genus *Cronobacter* consists of seven species: *C. sakazakii, C. turicensis s, C. malonaticus, C. condimenti, C. dublinensis, C. muytjensii*, and *C. universalis* [28,29]. The first three are the most frequently isolated from human infections [30]. The recently reclassified species are *C. zurichensis, C. helveticus, C. colletis*, and *C. pulveris* [31].

The use of essential oils (EOs) or natural plant-derived compounds as food additives could be one of the possible ways to control *Cronobacter* spp. in various types of food products. The use of natural essential oils inscribes well into the “clean label” trend and allows a reduction to some extent of the amounts of chemical preserving agents. The literature data on the susceptibility of *Cronobacter* genus bacteria to inhibitory effects of EOs are limited and they mainly concern the *C. sakazakii* bacteria species. There are insufficient scientific reports that have investigated the inhibitory effects of several EOs and carvacrol, thymol, eugenol, and cinnamic acid/trans-cinnamaldehyde against *C. sakazakii* and *C. malonaticus* [32]. The knowledge about the potential susceptibility of other species from the genus *Cronobacter* to EOs and active substances of plant origin is still very scanty.

The main goal of this research was to study the antimicrobial activity of bacterial cellulose produced by *Gluconacetobacter hansenii* ATCC 23769 and *Komagataeibacter* sp. GH1 soaked with oregano essential oil against strains representing the genus of *Cronobacter*. The indirect aim was to check whether BC with OEO could in the future be an effective material for the production of active food packaging that could extend the shelf life of some products by preventing the growing of *Cronobacter* bacteria.

## 2. Materials and Methods

### 2.1. Microbial Strains and Culture Conditions

Bacterial cellulose was obtained from strains of *Komagataeibacter* sp. GH1 isolated and genetically characterized by our scientific team [33] and *Gluconacetobacter hansenii* ATCC 23769, obtained from the American Type Culture Collection. Strains were grown on Hestrin-Scharmm (BTL, Lodz, Poland) medium which contains (amounts in gL^−1^): 20, D-dextrose; 5, yeast extract; 5, peptone; 2.7, Na_2_HPO_4_; 1.5, citric acid; pH 6.0 [34]. The cultivation was carried out under static conditions at a 28 °C for seven days. After this time, BC was taken from the liquid surface and dedicated to further research.

In this study, the tested microorganisms were five strains of the genus *Cronobacter*: *C. condimenti* s37, *C. muytjensii* s50, *C. sakazakii* lv27, *C. turicensis* lv53, and *C. malonaticus* lv31. Those strains were isolated by our team from market products of plant origin [35]. The tested strains were stored at −48 °C. Prior to use, they were grown freshly on a tryptone soya broth (TSB, Argenta, Poznan, Poland) according to reference [36].

### 2.2. Purification and Preparation of Biocellulose Discs

Obtained biocellulose films were washed with distilled water, and then incubated in 1% NaOH at 90 °C for 45 min to remove bacterial cells and medium components. After this stage, the films were neutralized with acetic acid and washed with distilled water [37]. The purified BC films were sterilized in distilled water at 121 °C for 20 min. After sterilization, BC films were dried at 23 °C under laminar chamber. Thicknesses of each sample of BC from two different strains of acetic bacteria were measured at 10 different positions, using thickness gauge. The results were expressed as the mean values of ten determinations of the BC membranes [38]. Discs with an 8 mm diameter were cut. Discs of filter paper also had an 8 mm diameter.

### 2.3. Impregnation of Bacterial Cellulose and Filter Paper with Oregano Essential Oil

As an antimicrobial agent for impregnating the biocellulose films, undiluted volumes of OEO was used (origanum oil, Sigma-Aldrich, Steinheim, Germany, W282812, 100% pure essential oil). For impregnation of BC, discs were immersed in 5 mL OEO for 24 h at 23 °C in a tightly closed container to prevent evaporation of the oil. The discs were then taken out and wiped in sterile filter paper to remove the non-absorbed oil. Pure BC discs without oregano essential oil were used as a negative control and sterile filter paper with OEO as a positive control [39]. The impregnation of the filter paper with OEO was carried out under the same conditions as the impregnation of BC.

BC discs after impregnation with OEO were weighed on an analytical balance (accurate to 0.0001 g, Radwag, Warsaw, Poland). OEO holding capacity was calculated by the formula (1) [40]:

OEO holding capacity (%) = (W_h_ − W_d_)/W_d_ × 100 (1)

where W_h_ is the weight of the hydrate BC disc and W_d_ is the weight of the dry BC disc; each measurement was carried out in triplicate.

### 2.4. Antimicrobial Activity of Bacterial Cellulose with Oregano Essential Oil

The antibacterial activity of BC impregnated with OEO was tested against five strains of *Cronobacter* sp. using the disc diffusion method. Bacterial inoculum was prepared from overnight culture on soybean agar with tryptone (TSA, Argenta, Poznan, Poland). Colonies were openly suspended in 0.85% physiological salt to obtain a turbidity comparable to the McFarland standard turbidity of 0.5 (approximately 8 log CFU mL^−1^). Aliquots (0.1 mL) were distributed over the surface of pre-dried tryptone soy agar plates using a sterile swabs. BC discs and discs of filter paper with an OEO and negative control without essential oil were placed onto plates with seeded bacteria and incubated for 24 h at 35 °C according to our previous methods [36], which was modified in this study. The diameters of the inhibition zones were measured in triplicate in millimeters using an electronic caliper. The measurement scale was as follows: strong inhibition zone of inhibition ≥ 20 mm (including disc diameter), moderate inhibition zone < 20–12 mm, and no inhibition zone < 12 mm. All analysis were carried out in triplicate. Valuesare presented as mean ± standard deviation.

### 2.5. Statistical Analysis

Results obtained were subjected to a statistical analysis using Statgraphics Plus software (Version 5.1, Warrenton, WV, USA). One-way (ANOVA) analysis of variance was done. Tukey’s test was used to compare the significance of differences between mean values (at a significance level of α = 0.05).

## 3. Results and Discussion

### 3.1. Oregano Essential Oil Holding Capacity

**Figure 1:**
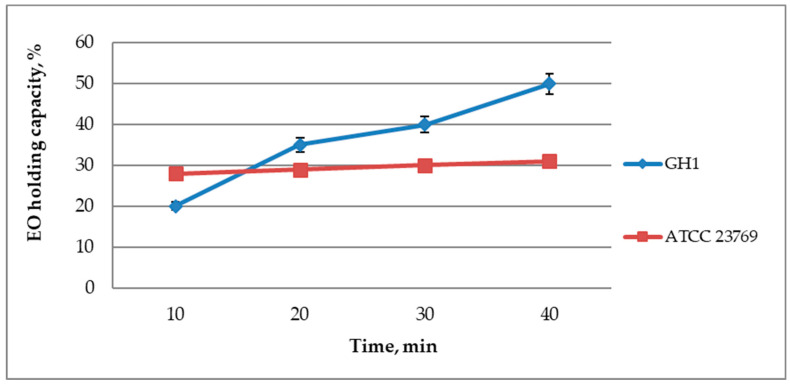
Oregano essential oil holding capacity. The data are presented as a mean ± standard deviation.

Bacterial cellulose has a nanoporous structure and has a high swelling ability, which allows saturating and releasing medicinal substances [41]. Therefore, we analyzed the ability of two kinds of biocellulose samples: one from *G. hansenii* ATCC 23769, and second from *Komagataeibacter* sp. GH1 to absorb and retain the oregano essential oil (Figure 1).

As a result of the work, it was determined that the BC films impregnated with OEO had good holding capacity. BC from the strain *Komagataeibacter* sp. GH1 was able to better absorb and retain OEO compared to BC produced by the *G. hansenii* ATCC 23769. Obtained results showed that BC from ATCC 23769 strain had less holding capacity, but it made this sample more predictable and consistent. It is likely that this difference in OEO holding capacity resulted from different arrangements of cellulose fibers as well as pore size and pore volume [42]. The differences in the OEO holding capacity could have been the consequence of differences in humidity during BC disc drying. BC samples were dried at different periods of time, although the same drying method was used, but no ambient humidity was measured. Based on the literature data, it can be assumed that this could have been a factor influencing the difference in holding capacity between BC obtained from ATCC 23769 and from GH1 strains [43,44].

### 3.2. Antimicrobial Activity of Bacterial Cellulose with Oregano Essential Oil

**Figure 2:**
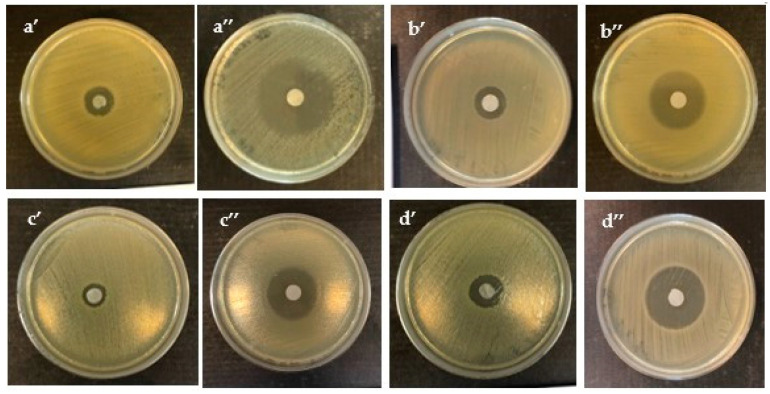
Inhibition zones against (**a**) *C. condimenti* s37, (**b**) *C. muytjensii* s50, (**c**) *C. turicensis* lv53, (**d**) *C. malonaticus* lv31, caused by BC discs with OEO produced by *G. hansenii* ATCC 23769 (**’**) produced by *Komagataeibacter* sp. GH1 (**’’**).

**Figure 3:**
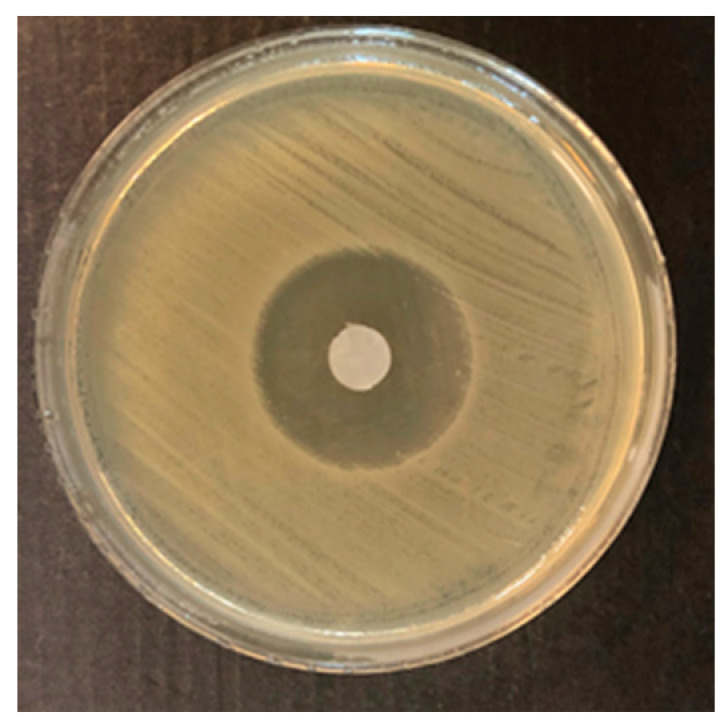
Inhibition zone against *C. malonaticus* lv31 caused by the filter paper disc with oregano essential oil.

The results of the antimicrobial activity of BC impregnated with the OEO by the disc diffusion method are shown in Figure 2 and Table 1. The inhibition zone against *C. malonaticus* lv31 caused by the filter paper disc with OEO was shown on Figure 3.

It was found that BC films and filter paper impregnated with OEO showed a strong and moderate inhibition zone of inhibition against all five *Cronobacter* strains and the diameter was from 13.6 ± 0.8 to 36.0 ± 0.7 mm. Filter paper showed definitely better inhibition properties of test strain growth in comparison with BC in the case of *C. condimenti* s37, *C. muytjensii* s50, and *C. sakazakii* lv27 (36.0 ± 0.7; 35.8 ± 0.9; 33.5 ± 1.5, respectively) (Table 1). However, in the case of *C. turicensis* lv53 as well as *C. malonaticus* lv31, the diameter of zone inhibitions caused by soaked filter paper was smaller than with using impregned BC obtained from *Komagataeibacter* sp. GH1 (Table 1). Similar antimicrobial activity of OEO in the paper disc diffusion assay was obtained against *Listeria monocytogenes*, *Salmonella typhimurium*, *Eschericha coli*, *Brochothrix thermosphacta*, and *Pseudomonas fluorescens*. It was shown that the zone of inhibition of *Salmonella typhimurium* was 14.1 ± 0.8 mm [45], which was two times less than that of *Cronobacter condimenti* 32.75 ± 2.8 mm BC of GH1 in our study. It was shown that BC produced by *Komogataeibacter* sp. GH1, impregnated with OEO, showed a strong antimicrobial effect against all *Cronobacter* strains from 16.39 ± 0.7 mm to 32.75 ± 2.8 mm and the inhibition zones were similar against *Enterococcus faecalis* 16.3 ± 0.09 mm, *Staphylococcus aureus* 26.8 ± 0.08 mm, *Staphylococcus epidermidis* 16.8 ± 0.09 mm, *Streptococcus mutans* 16.5 ± 0.07 mm, *Salmonella choleraesius* 29.0 ± 0.08 mm [46], and against *Citrobacter* spp. 24.0 ± 0.5 mm, *Salmonella Typhi* 22.4 ± 1.5 mm, and *Escherichia coli* 19.0 ± 2.2 mm [47].

Bacterial cellulose obtained from *Komagataeibacter* sp. GH1 soaked with oregano essential oils showed better inhibition properties in comparison with the bacterial cellulose obtained from *G. hansenii.* This fact is probably connected with the thickness of each biopolymer, which was in a range of approximately 30–45 ± 0.2 μm for *Komagataeibacter* sp. GH1 and about 20–28 ± 0.4 μm for *G. hansenii*. It was shown that the thickness of bacterial cellulose can be different for each strain, even while keeping the same cultivation conditions. This parameter depends on calcium source and general medium components, strain and methods of drying [38]. Despite the cultivation of acetic bacteria under the same conditions and for the same period of time, the thickness of the membrane varied. You-Jin et al. demonstrated that the longer the cultivation time, the thicker the cellulose membrane and the better the absorption properties, so in the future, the cultivation time should be lengthened to obtain a more inhibitory effect [48].

The essential oils extracted from aromatic plants have bioactive components in their chemical composition, for example, monoterpenes, monoterpenoids, and phenylpropanoids. Their concentrations in the extracted oils depend strongly on the soil, climatic, and geographical conditions of the herbal culture growing. The study developed on hydroxyapatite coated with peppermint essential oil showed strong antimicrobial properties of this material against *S. aureus* (including the methicillin-resistant strain), *E. faecium*, and also *P. aeruginosa* [49].

Based on the obtained results, it can be concluded that the biologically active components of OEO can exhibit antimicrobial activity against several pathogenic bacteria. The largest diameter of the inhibition zone was observed in all samples with OEO against the *C. condimenti* s37 strain. However, filter paper as a carrier had higher inhibition zones compared to BC, but it should be taken into account that BC has higher mechanical strength compared to plant cellulose, so it is worth using this biomaterial in further research [50]. It was noted that cellulose type Iα has a stronger structure than cellulose Iβ. In bacterial cellulose, the type Iα content is approximately 60%, while that in cotton is 30%. BC has the best water-holding ability of 98%, while plant cellulose is 60% [51].

Although the filter paper soaked in OEO showed greater antimicrobial activity, the aim of the research undertaken was to strive to develop an active packaging prototype based on natural polymer. The research described in this manuscript provides valuable information that it is possible to soak bacterial cellulose with a natural biologically active substance and such a combination has a strong effect on inhibiting the growth of *Cronobacter* bacteria. BC is insoluble in water, unlike traditional paper, and has a definite advantage in mechanical strength. This is sufficient argument to continue work on the use of BC with OEO as active biodegradable packaging intended for contact with food.

## 4. Conclusions

Based on this study, it was concluded that bacterial cellulose impregnated with oregano essential oil has strong and moderate antimicrobial activity against all presented strains of the genus *Cronobacter* isolated from plant matrix. Bacterial cellulose of strain *Komagataeibacter* sp. GH1 was a better carrier of oregano essential oil as it has the best antagonistic activity compared to bacterial cellulose obtained from *G. hansenii* ATCC 23769 and strength compared to filter paper. Based on this conclusion, bacterial cellulose of strain *Komagataeibacter* sp. GH1 can be further investigated as food packaging to improve the safety and shelf-life of food.

Based on these results, it is worth providing similar research based on antimicrobial properties of bacterial cellulose with oregano essential oil. The next stage of research will concern the design of active packaging based on bacterial cellulose with oregano oil and carrying out load tests on the example of ready-to-eat vegetable lettuce. The obtained results give hope that in the future, it will be possible to replace synthetic materials (which are not environmentally friendly) with biodegradable materials such as bacterial cellulose enriched with active substances.

## Figures and Tables

**Table 1:** Inhibition zone diameter and mean ± SD (mm) using the disc diffusion method.

Cronobacter Strains	BC with OEO Produced by *G. hansenii* ATCC 23769	BC with OEO Produced by *Komagataeibacter* sp. GH1	Filter Paper
*C. condimenti* s37	14.7 ^a^ ± 0.8	32.75 ^b^ ± 2.8	36.0 ^b^ ± 0.7
*C. muytjensii* s50	14.5 ^a^ ± 0.5	17.23 ^b^ ± 0.8	35.8 ^c^ ± 0.9
*C. sakazakii* lv27	14.1 ^a^ ± 0.4	16.39 ^a^ ± 0.7	33.5 ^b^ ± 1.5
*C. turicensis* lv53	13.6 ^a^ ± 0.8	18.19 ^b^ ± 0.3	30.5 ^c^ ± 0.5
*C. malonaticus* lv31	14.3 ^a^ ± 0.5	28.33 ^b^ ± 2.6	27.1 ^b^ ± 0.7

^a, b, c^ means with different letters in a line are significantly different (*p* < 0.05, *n* = 3).
